# Facts, Not Forebodings

**Published:** 1895-10

**Authors:** 


### EDITORIAL.

### FACTS, NOT FOREBODINGS.

THE open letter from the pen of one of the most prominent veterinary surgeons in America, which we print on another page of this issue, is as startling in its language as it was unexpected and uncalled for from such a source; and while it fell flat and helpless before the body at whom it was directed and destined, and only mingled words of pity and sorrow for its author were uttered, still we do not feel as a public journal that we would be doing our duty to the cause we uphold, nor to our readers, to allow such an issue of misstatements and innuendoes to go unchallenged. Tempered as must be the reply of the writer because of parental relations and veneration for one to whom he feels so greatly indebted for the honorable services he so long performed on behalf of the profession, but which promises now to be clouded in the evening of his life by what may be generously construed as errors of judgment, not of heart, still the cause of higher veterinary education must go on and the triumphs of the past of bodies drawn together by a common purpose, cemented only by a single desire to attain a given end in the welfare and interests of a class of people; organizations that need no legal or constitutional laws to bind them together have ever been the strongest bodies in the history of America, and the day of their weakness and decay will dawn when they must seek for *legal* and *constitutional laws* and *usages* to maintain their aims and purposes.

Ostensibly written in a foreign country, but printed in large numbers for American distribution, and designed to check and retard one of the grandest movements ever inaugurated in America, and reaching its place at an hour and time when this movement was receiving the highest honor and riches it could hope to obtain, by the presence of the largest representation of veterinary institutions of America, it seemed well that it should at this time have emerged from its place of concealment, for its language and statements could there be considered and refuted before the assemblage whose interests and purposes it assailed.

For many years the writer had the unqualified and unceasing support of the author of that letter in his efforts dating back ten years ago to bring together the veterinary educational institutions of America in a common purpose to advance veterinary education in our country, and none seemed more disappointed at the failures than the author referred to, so much discouraged at the prospects of success, that we believe that the one prime reason why the school he represents was not present in Buffalo at 1893 was largely because he felt that this final movement was only destined to be another failure, and it was not worth the effort and expense. But let it be known now, once for all, the Association of American Veterinary Faculties has come to stay; will grow stronger day by day; will outlive the charges and attacks and calumnies of any one man; will not stop, will not dissolve, will not begin over again, needs no legal or constitutional fetters to bind it together—for higher, better, and stronger than all these are the honor, the motives, the purposes and aims of the men who compose it, and the objects to be attained will be grander and more honorable because it is thus.

When we answer the chief interrogatory of the whole letter, by what authority does this organization exist? We reply by the same authority that the United States Veterinary Medical Association exists; the common purpose, desires, aims, and determination of strong men to do something more for their calling besides existing upon its fruits, as does the parasite upon its prey. And more the same determined purposes of that body that has made it an honored institution in our land, admired abroad by our colleagues, and respected to-day by every veterinarian in America, followed and fashioned after by every interstate, State, city, and county organization; all of whom are to-day loaning their influence, recognition, and support to this Association of Faculties, is why it lives and will grow better and stronger every year in the future.

Why does the author of that letter insult the children of the school he represents when he refers to a telegram which he says was sent to *his friend*, Prof. Robertson? Who is Prof. Robertson, I ask? Is he Prof. Jas. L. Robertson, who has so well and honorably filled the Chair of Theory and Practice in the American Veterinary College? And if so, was he not an accredited representative of that College? Did he not comply with the requirements of the organization and sign its membership-list? Was he not qualified to speak for that school? If not, who was? Are her children to realize at this late day that no one can speak for that school save the author of that letter? If so, then why is the question propounded asking for reasons why the Board of Trustees, Faculty, Officers, etc., were not notified of this proposed movement?

As chairman of the committee delegated to accomplish the organization of this Association, I reply that there is not a school in North America whose designated head but what was notified more than once of the meeting in Buffalo, and before its date was fixed was consulted as to a fitting time and place; and the answers of these schools of North America are in the possession of the writer of this reply, to be produced whenever required before any properly delegated body. Now if these delegated heads of colleges did not deem it of sufficient importance to bring these communications before their boards of officers or trustees, or if, as we are allowed to imply, it was not necessary in some cases, why then will the author of that letter boil over with indignation at the thought that the Board of Trustees of the school he represents were not consulted, and then ignores one of the foremost of his faculty colleagues by referring to him as his friend.

Let it not be forgotten that there has not a single veterinary college opened its doors since the inauguration of this movement but what has complied with the sense of this Association in believing that nothing less than a three-years' course of six months each should be the minimum standard. Let it be remembered to the honor of this Association and its parent, the United States Veterinary Medical Association, that this year dates the last of a two-years' course in two of the most prominent veterinary schools in this country, and that these are the convictions of almost every other of the two-year schools.

Let it be remembered that all the laws and State boards of veterinary examiners created since the organization of this Association of Faculties have, in compliance with the sentiment of that body, fixed a three-years' course of six months each as the minimum of requirements that will entitle their future graduates to examination before said boards.

